# Decrease of activity of antioxidant enzymes, lysozyme content, and protein degradation in milk contaminated with heavy metals (cadmium and lead)

**DOI:** 10.3168/jdsc.2022-0222

**Published:** 2022-07-22

**Authors:** G. Grassi, A. Simonetti, E. Gambacorta, A. Perna

**Affiliations:** School of Agricultural, Forestry, Food and Environmental Sciences, University of Basilicata, Potenza, Viale dell'Ateneo Lucano 10-85100, Italy

## Abstract

The aim of this study was to evaluate the effect of added Cd and Pb to milk on its stability by determining antioxidant enzymatic activities, lysozyme content, and protein degradation. Antioxidant enzymatic activities were spectrophotometrically determined by superoxide dismutase, catalase, xanthine oxidase, and glutathione peroxidase assays; lysozyme was identified and quantified by HPLC-UV analysis, and protein degradation was investigated by spectrophotometric analysis of advanced oxidation protein products (AOPP) and dityrosine content. In this study, contaminated milk samples showed a significant reduction in activity of all studied enzymes compared with control milk. The contamination of milk also led to a significant reduction in the lysozyme content; lysozyme content was decreased about 22% and 18% in Pb milk and Cd milk, respectively, compared with control milk. The presence of the contaminants in the milk resulted in a significant increase of both dityrosine concentration and AOPP compared with the control milk. Moreover, between types of contaminated milk, dityrosine and AOPP values were significantly higher in the Pb milk than in the Cd milk. Therefore, it is important to monitor the presence of these toxic elements in milk for the damage they cause to consumer health both directly due to their ingestion and indirectly due to loss of milk stability.

## Highlights

• Cadmium and lead in milk influenced the oxidative stability of protein.
• Contaminated milk showed a significant reduction in activity of all studied enzymes.
• The milk contamination led to a significant reduction in the lysozyme content.
• Contaminated milk resulted in an increase of dityrosines and AOPP.
• Dityrosine and AOPP values were higher in the Pb milk than in the Cd milk.

## Graphical Abstract

**Figure:**
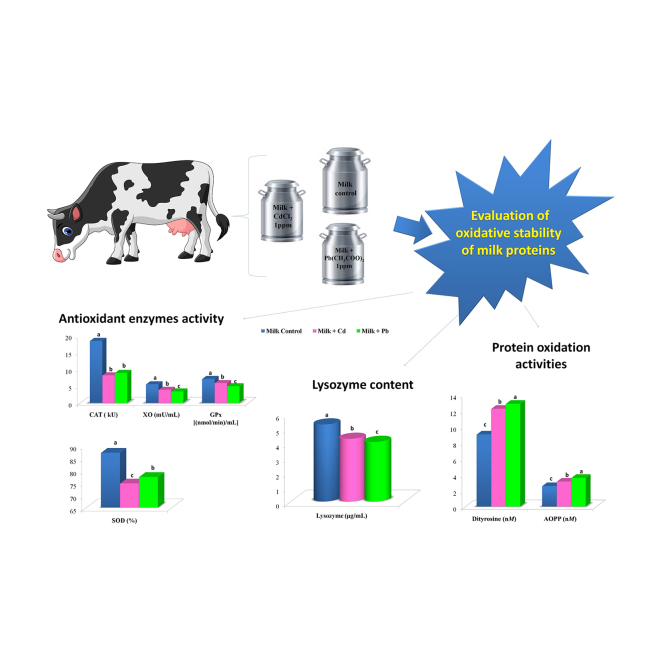
**Summary:** The addition of cadmium (Cd) and lead (Pb) to the bulk milk of Friesian cows resulted in a significant change in its oxidative stability. The activity of endogenous antioxidant enzymes such as catalase (CAT), xanthine oxidase (XO), glutathione peroxidase (GPX), and superoxide dismutase (SOD) was significantly lower in contaminated milk. The presence of the toxic heavy metals significantly reduced the lysozyme content, whereas the protein oxidation compounds, dityrosine and advanced oxidation protein products (AOPP), increased compared with the control milk. Comparing the effect of the 2 metals, it was found that the Cd caused a more marked inhibition of the activity of CAT and SOD, while Pb inhibited the activity of XO and GPX more. Furthermore, compared with the control milk, the lysozyme content was lower in the Pb milk and the dityrosine and AOPP values were significantly higher in the Pb milk than in the Cd milk.

Environmental contamination by heavy metals is a growing concern due to the adverse health effects. Among heavy metals, lead (Pb) and cadmium (Cd), the two most present, often coexist in a polluted environment (Ozmen and Mor, 2004). Concern about these toxic elements also stems from the numerous routes of exposure. The most common sources of contamination are contaminated water, lead paint, car emissions, industrial emissions, and mining extractions. The food chain is one of the most important causes of Cd and Pb accumulation because the abundance of these heavy metals in the atmosphere creates a direct connection with the distribution in the chain soil–cattle feed–milk (Vidovic et al., 2005).

Significant quantities of Pb and Cd can be transferred from contaminated soil, plants, and grass to grazing animals (Miranda et al., 2005). Ingestion of these elements causes toxic effects to the animals through direct contact, and to humans who consume contaminated meat and milk (Cai et al., 2009). In fact, milk is an excretion of the mammary gland and as such can carry numerous xenobiotic substances including toxic metals. The high content of Cd and Pb in milk could pose a public health problem (Swarup et al., 2005). Many authors have highlighted the presence of Cd and Pb in land used for both livestock grazing and to grow crops for animal feed (Nabulo et al., 2011). At the early stages of an animal's life, the intake of heavy metals could cause harmful effects such as reduced developmental performance, neurotoxicity, oxidative stress, cell death, and immunotoxicity (Santos et al., 2018).

Oxidative stress is one of the main toxic effects linked to the mechanism of action of Pb and Cd. However, the mechanisms involved in this effect are still unclear and are the subject of ongoing research. It is known that the body's defense mechanism is directly related to the effects of antioxidant activity of specific enzymes such as superoxide dismutase (**SOD**), catalase (**CAT**), glutathione peroxidase (**GPx**), and glutathione transferase (**GST**; Gusti et al., 2021). Dalle-Donne et al. (2008) showed that limited exposure to toxic metals improved the enzymatic performance, specifically the SOD, CAT, GPx, and glutathione reductase activities; this could be linked to the cell's ability to adapt and activate defense mechanisms to counteract oxidative action. Conversely, studies have shown that when exposure to Pb and Cd lasts longer, the activity of antioxidant enzymes in cells drastically decreases due to the displacement of Mn, Cu, and Zn ions from the active site of MnSOD and CuZnSOD, Fe ions from catalase, or Se ions from glutathione peroxidase (Dalle-Donne et al., 2008; Patra et al., 2011). In addition, Cd and Pb modify the composition of fatty acids of the cell membrane and promote the synthesis of reactive oxygen species (**ROS**; Singh et al., 2019). Cadmium and Pb also showed a high affinity with the sulfanyl groups (−SH) of proteins, such as GSH, GPx, and CAT, affecting the molecule functionality and, therefore, their antioxidant activity (Ercal et al., 2001). Thus, the aim of this study was to evaluate, as a first step, the effect of direct milk contamination with Cd and Pb on activity of antioxidant enzymes and lysozyme content, and protein degradation of milk.

Milk from Italian Holstein cows reared indoors on farm in the province of Potenza (southern Italy) was used in this experiment. Animal care and use approval was not necessary because the study was conducted on bulk milk purchased at the company, authorized for direct milk sales. For the study, the milk of the morning milking was collected to make a composite sample: after the milk was divided into 3 batches of 1 L each: in the first lead acetate [Pb(CH_3_COO)_2_; Sigma-Aldrich] was added to obtain a final concentration of 1 ppm, in the second cadmium chloride (CdCl_2_; Sigma-Aldrich) was added to obtain a final concentration of 1 ppm, and the third was milk control (without any addition). Sampling was done 6 times. The milk samples were stored at a refrigeration temperature (4°C) for 24 h in glass containers and analyzed for evaluation of antioxidant enzyme activity, lysozyme content, and protein oxidation activities.

Before carrying out the enzyme activity assays, the pH value of each milk sample was adjusted to 4.6 with HCl 0.1 *M*. After that the samples were centrifuged at 5,000 × *g* for 20 min at 4°C, and NaCl 0.1 *M* was added to recovered supernatants up to pH 6. The supernatants were filtered through a Whatman no. 40 filter paper and kept at −20°C until analysis. All measurements were made in triplicate.

The SOD activity was detected by measuring the inhibition of pyrogallol autoxidation following the method proposed by Li et al. (2018), with some modifications. Briefly, the reaction mixture was prepared by adding 1.9 mL of Tris-HCl 0.1 *M*, 50 μL of sample, and 50 μL of pyrogallol 20 m*M* in HCl 1 m*M*. Inhibition of self-oxidation was followed by spectrophotometrically (UV-VIS Spectrophotometer1204; Shimadzu) at 325 nm every 30 s for 3 min. The results were expressed as percentage inhibition [I (%)] and were calculated by the following equation:

I (%) = [A_b_ − (A_s_ − A_t_)/Ab] × 100,

where A_b_ = absorbance of blank sample (sample was replaced by solvent; t = 3 min); A_s_ = absorbance of sample (t = 3 min); and A_t_ = absorbance of the test sample (pyrogallol was replaced by distilled water; t = 3 min).

The CAT activity was assessed in accordance with the method described by Hadwan and Abed (2016). The absorbance of the complex was measured at 374 nm (UV-VIS Spectrophotometer 1204; Shimadzu) against blank and the first-order reaction rate constant (k) equation was used to determine catalase activity, as follows:

$$kU=\frac{2.303}{t}\times \left[log\frac{S^{\circ }}{S-M}\right]\times \frac{V_{t}}{V_{s}},$$

where t = time (3 min); S° = absorbance of standard (without sample); S = absorbance of sample; M = absorbance of the control test (without hydrogen peroxide; correction factor); V_t_ = total volume (5,100 µL); and V_s_ = volume of serum (100 µL).

Xanthine oxidase (**XO**) activity was evaluated by a spectrophotometric assay as described by Durak and Öztürk (2014). The reaction mixture was prepared by adding 2.7 mL of 46.7 m*M* buffer phosphate (pH 7.5); 100 μL of 0.17 m*M* xanthine; and 100 μL of sample heated at 37°C. The mixture was incubated at 37°C for 30 min; after that, 100 μL of trichloroacetic acid 3.33% (wt/vol) was added and centrifuged at 5,000 × *g* for 10 min at room temperature. The absorbance was measured at 293 nm and the activity was calculated by the following equation:

$$\frac{U}{mL}=\left(\frac{A_{s}-A_{b}}{min}\right)\times \frac{1}{\varepsilon }\times \frac{V_{T}}{V_{S}}\times \frac{1}{1,000mL},$$

where A_s_ = absorbance of sample; A_b_ = absorbance of blank (trichloroacetic acid addition before incubation to stop the reaction); ε = uric acid (obtained by conversion reaction of XO) extinction coefficient; V_T_ = total volume (3 mL); and V_S_ = volume of sample (0.100 mL).

For GPx activity, a spectrophotometric method was conducted as described by Torres et al. (2003). The absorbance was measured at 340 nm for 2 min, using a thermostated spectrophotometer at 37°C. Oxidized NADPH (nmol/min per mL) in samples to which enzymatic activity is directly related were calculated as follows:

GPx(nmol/nmolmin*minmL)=(ΔA340/ΔA340min*min0.00373M−1)×VrVs×Ds,

where ∆A_340_/min = change in absorbance per minute of samples; V_r_ = total volume (0.19 mL); V_s_ = volume of sample (0.02 mL); D_s_ = sample dilution; and 0.00373 *M*^−1^ = the extinction coefficient of NADPH.

Lysozyme extraction and quantification were carried out as described by Matera et al. (2022). The analysis was performed in liquid chromatography equipped with Varian ProStar Pump model 210, Rheodyne injector with a 20-mL loop, UV-VIS detector Varian ProStar model 325, and Galaxie Chromatography Software (Varian Inc.) using a C18ViVa column (5 μm, 4.6 mm, 150 mm; Restek, USA). The mobile phase consisted of trifluoracetic acid (1 mL/L in deionized water; eluent A) and 1 mL/L trifluoracetic acid, 950 mL/L acetonitrile in deionized water (eluent B). The injection volume for all samples was 20 µL. The elution was with flow rate 1.0 mL∙min^−1^, and the gradient elution was as follows: 100% solvent A for 5 min followed by a linear gradient to 50% B (vol/vol) over 15 min, increasing to 60% B (vol/vol) over 5 min and running at 60% B (vol/vol) for 10 min. The eluted protein was monitored at 280 nm, the standard solution of lysozyme from chicken egg (Sigma-Aldrich; 5–100 µg/mL) was used for identification and quantification of the peaks, and the results were expressed as micrograms per milliliter of milk.

Advanced oxidation protein products (**AOPP**) and dityrosine in milk samples were determined measured by spectrophotometric method. The AOPP were determined as described by Witko-Sarsat et al. (1996), using a calibration curve with chloramine-T standard solution (Sigma-Aldrich; 0–100 µ*M*). The absorbance was measured at 340 nm and AOPP concentrations were expressed in n*M* chloramine-T equivalent. Dityrosines were determined as described by Witko-Sarsat et al. (1996). The absorbance was measured at 315 nm and dityrosine concentration was determined using the Lambert-Beer formula (ε = 5 m*M*^−1^∙cm^−1^, pH 7.5).

Statistical analysis was performed using the general linear model procedure of SAS (1996, SAS Institute Inc.) using a monofactorial model. Differences among contaminated milk were analyzed using Student's *t*-test, and differences between means at the 95% (*P* < 0.05) confidence level were considered significant.

In this study, the Cd and Pb contents in milk samples without any addition were 6.32 ± 0.58 and 12.78 ± 1.16 ppb, respectively; these values were lower than the allowed limits announced by the standard organizations. The activity of antioxidant enzymes in milk samples with and without contaminants is shown in Table 1. The contamination significantly influenced the studied parameters (*P* < 0.001); contaminated milk samples showed a significant reduction of enzyme activity compared with control milk. Superoxide dismutase is an enzyme capable of removing ROS by catalyzing its dismutation to O_2_ plus H_2_O_2_ (Fattman et al., 2003). In contaminated milk the SOD activity significantly decreased (*P* < 0.01) resulting in an inhibition of activity of 14.22% and 11.20% in Cd milk and Pb milk than in control milk. In support of our results, Zhang et al. (2014) found a significant inhibition of SOD activity in zebrafish liver with higher Pb concentrations. Several studies detected a lower SOD activity in erythrocytes of both Pb-treated calves (Swarup et al., 2005; Patra et al., 2011) and Pb-treated rats (Annabi Berrahal et al., 2007) compared with the control group. On the contrary, Shi et al. (2005) reported an increase of SOD activity in the liver of *Carassius auratus* after 24 h of Cd exposure (5 mg/L), and Gupta et al. (2009) found elevated SOD activity in roots and shoots of *Zea mays* seedlings after Pb exposure. Alghazal et al. (2008) considered that SOD activity undergoes upregulation as a function of Pb oxidative stress (compensatory response). Catalase is the enzyme that best expresses its action as a catalyst promoting the change of hydrogen peroxide into water and molecular oxygen (Nandi et al., 2019). Catalase activity was significantly higher in control milk, which showed the highest reaction rate compared with contaminated milk (*P* < 0.01; Table 1). The presence of contaminants in milk resulted in a percentage inhibition of activity higher than 50% (53.74% and 55.53% in Cd milk and Pb milk, respectively).

**Table 1:** The activity of antioxidant enzymes in milk samples with and without contaminants

Item^1^	Milk control		Milk + Cd		Milk + Pb	
	Mean	SD	Mean	SD	Mean	SD
SOD (%)	87.04^a^	7.42	74.66^c^	3.75	77.29^b^	2.73
CAT (kU)	18.40^a^	2.81	8.16^b^	1.39	8.89^b^	1.48
XO (mU/mL)	5.51^a^	0.47	3.87^b^	0.33	3.39^c^	0.30
GPx [(nmol/min)/mL]	7.10^a^	1.27	5.86^b^	1.12	4.93^c^	1.07

a–c Different lowercase superscripts depict a significant difference within a row (*P* < 0.01).

1 SOD = superoxide dismutase; CAT = catalase; XO = xanthine oxidase; GPx = glutathione peroxidase.

Similar results were found by Shi et al. (2005), who observed a inhibition of CAT activity in the liver of *C. auratus* after 24 h of Cd exposure (5 mg/L). An increase in ROS results in a change in activity of the CAT and SOD enzymes due to the need to decompose these enzymes to preserve the balance of the cell's intrinsic antioxidant defenses. Zhang et al. (2014) hypothesized that changes in CAT and SOD activity are directly related to the interaction of Cd and Pb with the enzyme molecule, which can cause both misfolding and alteration of enzyme activities. Wang et al. (2015) found that Cd through electrostatic force binds to substrate channels, subsequently entering the active sites of CAT and SOD. In line, Zhang et al. (2014) reported that Pb could make complexes with SOD penetrating the active channel of SOD and hindering access to the substrate. The metal thus interacts with Arg 141, an enzymatically related amino acid residue, modifying the secondary structure of SOD and resulting in the release of Cu^2+^ and Zn^2+^ from the active site of SOD, with subsequent decrease of enzymatic activity. Xanthine oxidase is a powerful enzyme that stands in defense of the cell to counteract the oxidative stress caused by ROS and reactive nitrogen species. It produces uric acid that has a high antioxidant capacity, counteracting the negative action of free radicals. The XO, therefore, covers the role of protective regulator of the cellular redox potential for its dual action of both synthesis of uric acid and antioxidant (Valko et al., 2007). The XO activity underwent a reduction of about 34.12% in the contaminated milk compared with the control (*P* < 0.01); in particular, the reduction was more pronounced in Pb milk compared with Cd milk: 38.48 and 29.76%, respectively. The same behavior was observed for GPx, a selenium-dependent enzyme that reduces H_2_O_2_ and other peroxides at a high rate. The Pb milk showed almost twice as much inhibition of activity as the Cd milk (30.56 and 17.46%, respectively). In support of our results, Ikediobi et al. (2004) found that in rat Cd-stressed liver cells, the enzymatic activity of SOD, CAT, GPx, and glutathione reductase was reduced over a 4-h exposure to Cd^2+^ concentrations ranging from 100 to 300 µ*M*. It is known that heavy metals are elements that affect the catalytic activities of the enzyme (Hinojosa et al., 2004); Cd and Pb have high affinity for thiol groups (−SH) present in enzymes, inhibiting their activity (Nair et al., 2013).

Recent studies have shown the presence of phosphorylated binding sites in the molecule of some antioxidant enzymes, such as xanthine oxidoreductase, which could increase the affinity especially with Pb (Henry et al., 2015). Moreover, many authors detected the antagonism between Cd and Pb and some trace elements that have an important functional role being cofactors of many enzymes, such as zinc, magnesium, and selenium (Matović et al., 2012). Cadmium and lead are bivalent cations and tend to replace these elements, leading to the inactivation of the enzyme itself, with loss of antioxidant function. The marked reduction of CAT detected in the present study in contaminated milk could also be due to the interaction of metals, particularly Cd, with iron (Fe) present in the heme group of the enzyme (Mylroie et al., 1984). Cadmium and lead showed a high affinity with the −SH of proteins; this influences the functionality of the molecule and, therefore, the antioxidant activity of the enzymes (GPx, GSH, and CAT) that use a −SH group as a hydrogen donor (Ercal et al., 2001). Lysozyme is an enzyme naturally present in animal tissues with bactericidal properties by lysing the cell wall of bacteria (Jash et al., 2015). The milk contamination also led to a significant reduction in the lysozyme content (*P* < 0.001; Table 2), which adversely affects the bactericidal capacity of lysozyme, thus making milk more susceptible to bacterial alterations. In terms of percentage decrease, compared with control milk it was more than 22% and 18% in Pb milk and Cd milk, respectively. Olmo et al. (2001) and Zhang et al. (2013) observed that Cd and Pb can form complexes with lysozyme with conformational changes and a reduction in molecule size. Wang et al. (2016), thanks to a study conducted on the interaction between CdCl_2_ and lysozyme content, showed that Cd binding mechanisms are driven by hydrophobic forces. In particular, the above mentioned study, thanks to the use of biophysical methods, dynamics simulation, and measurements of enzymatic activity, highlighted how CdCl_2_ interacted directly with amino acid residues in the lysozyme chain, causing conformational changes and thus its degradation. The measurement of protein oxidation products is an index of oxidative stress in milk (Lindmark-Månsson and Åkesson, 2000). The oxidation of proteins is one of the main responses to a chemical alteration. However, the oxidation of protein components is a less studied phenomenon than lipid oxidation, but is noteworthy as it is linked to the loss of the nutritional value and organoleptic properties of the food; moreover, the intake of the resulting catabolites can have deleterious consequences on human health (Estévez and Luna, 2017). The oxidation of proteins generated by radicals can lead to damage to the protein skeleton or side chains and the oxidative damage of proteins is irreversible due to a slight unfolding and loss of function (Celi and Gabai, 2015). In this regard, in this study 2 markers were considered: dityrosines, components of protein oxidation resulted from the interaction of 2 tyrosine molecules (Fuentes-Lemus et al., 2018), and the AOPP, which determine the formation of reactive species with an increase in protein oxidation. As shown in Table 2, the presence of the contaminants in the milk resulted in a significant increase of both dityrosine concentration and AOPP compared with the control milk (*P* < 0.01). Furthermore, among the contaminated milk, the values of dityrosine were significantly higher in the Pb milk compared with the Cd milk (*P* < 0.01), which compared with the control increased by 28.22% for Pb milk and by 26.29% for Cd milk. The presence of contaminants in milk also determined a percentage increase compared with the control of AOPP, equal to 40.23% for Pb milk and 21.88% for Cd milk.

**Table 2:** Lysozyme content, dityrosine concentration, and advanced oxidation protein products (AOPP) in milk samples with and without contaminants

Item	Milk control		Milk + Cd		Milk + Pb	
	Mean	SD	Mean	SD	Mean	SD
Lysozyme (μg/mL)	5.24^a^	0.15	4.26^b^	0.18	4.05^c^	0.17
Dityrosine (n*M*)	9.03^c^	0.29	12.25^b^	0.37	12.88^a^	0.24
AOPP (n*M*)	2.56^c^	0.16	3.12^b^	0.10	3.59^a^	0.14

a–c Different lowercase superscripts depict a significant difference within a row (*P* < 0.01).

The aim of this study was to highlight the effect of Cd and Pb on the oxidative stability of milk proteins. Contaminated milk showed a significant reduction of both antioxidant enzyme activities and lysozyme content; moreover, the presence of Cd and Pb in the milk resulted in a significant increase of protein oxidation compounds such as dityrosine concentration and AOPP. Therefore it is important to monitor the presence of these toxic elements in milk for the damage they cause to the consumer health both directly due to their ingestion and indirectly due to loss of milk stability.
